# Malignant pleural mesothelioma with resolution of pleural effusion

**DOI:** 10.1002/rcr2.1234

**Published:** 2023-10-15

**Authors:** Sayaka Nishida, Kazutoshi Toriyama, Makiko Yomota, Yukio Hosomi

**Affiliations:** ^1^ Clinical Resident Tokyo Metropolitan Cancer and Infectious Diseases Center Komagome Hospital Tokyo Japan; ^2^ Department of Thoracic Oncology and Respiratory Medicine Tokyo Metropolitan Cancer and Infectious Diseases Center Komagome Hospital Tokyo Japan

**Keywords:** asbestos, malignant pleural mesothelioma, pleural effusion

## Abstract

In malignant pleural mesothelioma patients, pleural effusion may improve during the course of the disease. Pleural effusion with nodular shadows bordering the pleura should be followed up even if the pleural effusion improves.

## Graphical abstract

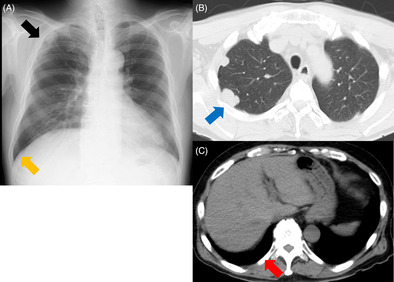

## CLINICAL IMAGE

**FIGURE 1:**
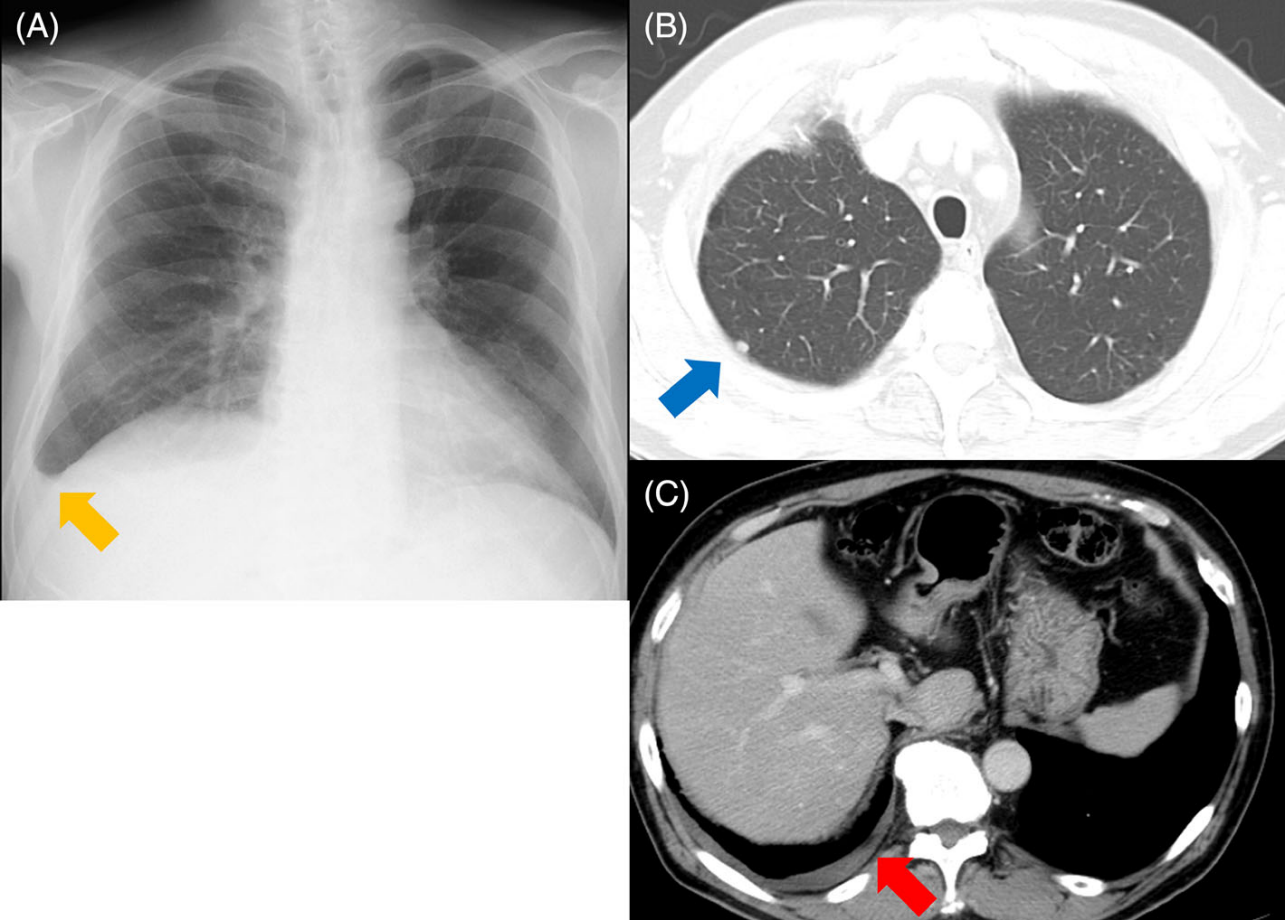
Chest x‐ray showed right pleural effusion (yellow arrow) (A). Chest computed tomography showed a nodular shadow bordering the pleura (blue arrow) (B) and right pleural effusion (red arrow) (C).

**FIGURE 2:**
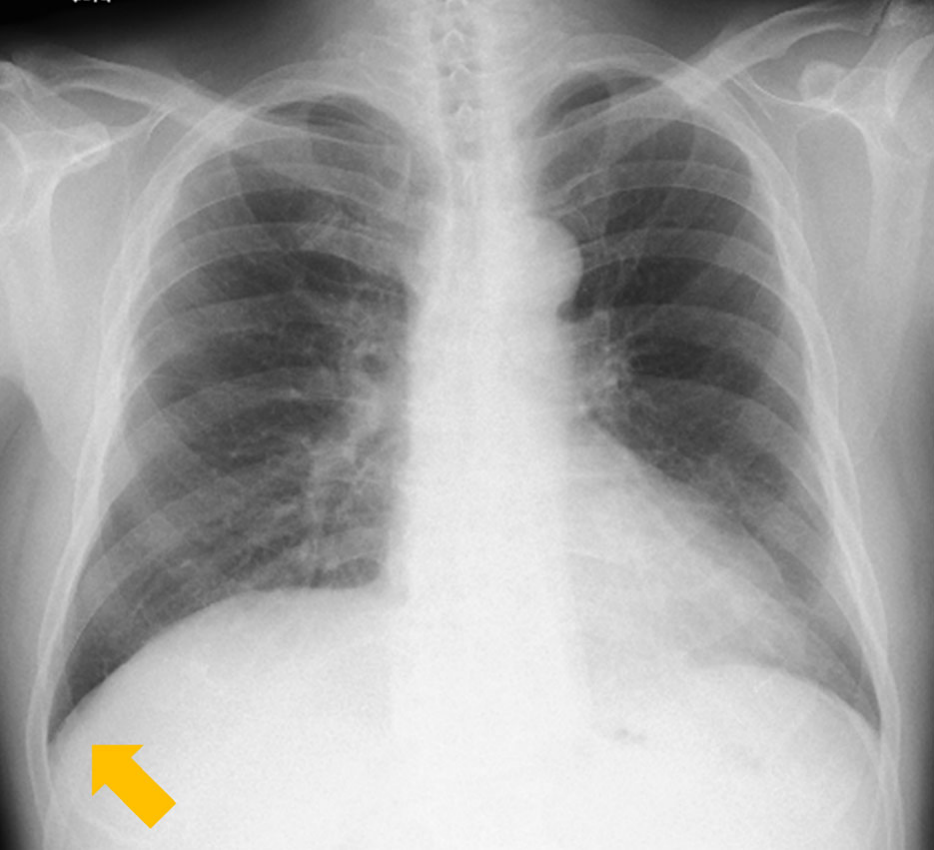
Chest x‐ray showed no pleural effusion (yellow arrow).

**FIGURE 3:**
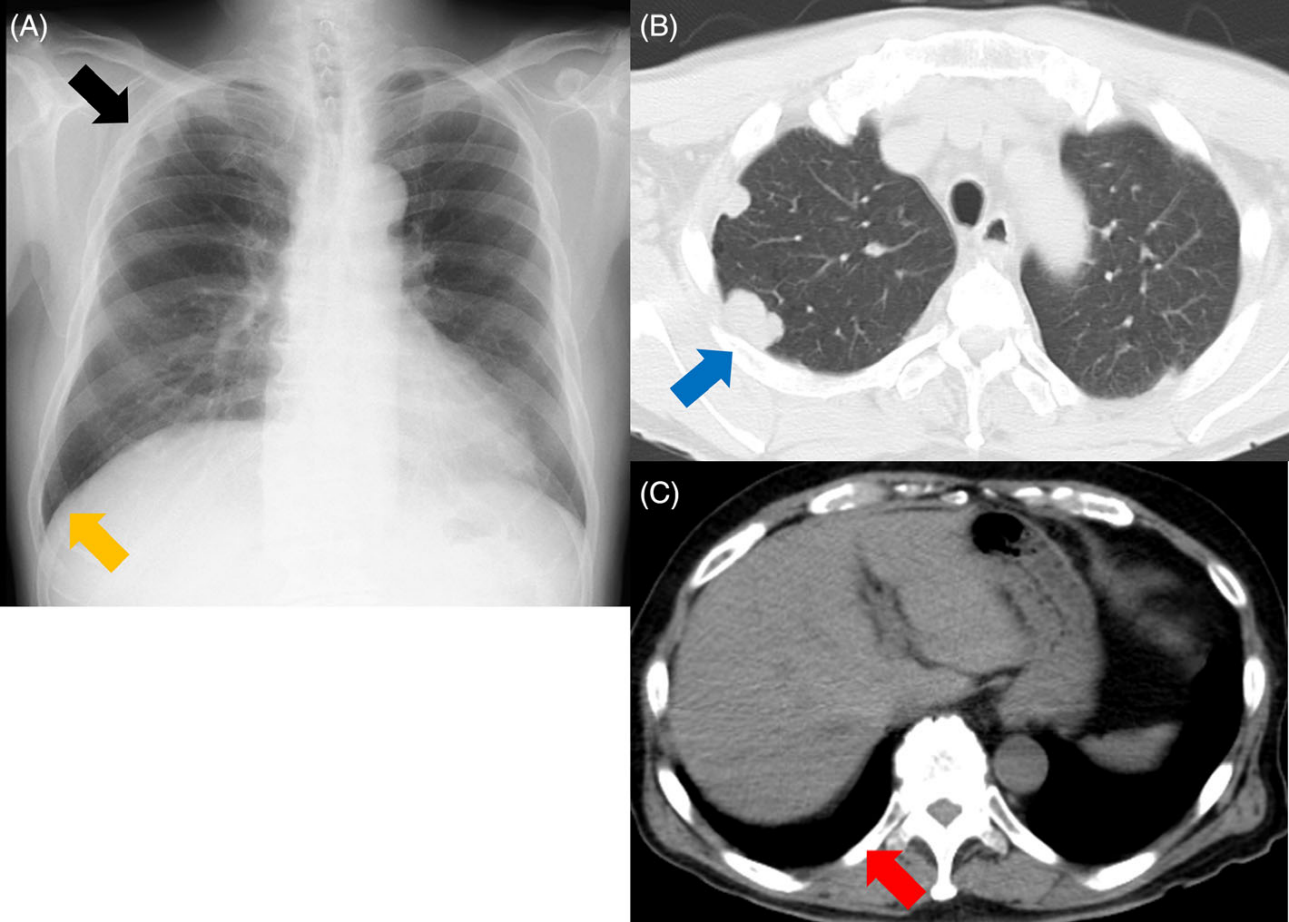
Chest x‐ray showed an abnormal shadow in the right upper pulmonary lung field (black arrow) but did not show pleural effusion (yellow arrow) (A). Chest computed tomography showed a nodular shadow bordering the pleura (blue arrow) (B) but did not show pleural effusion (red arrow) (C).

A 69‐year‐old, male patient visited our hospital 4 years ago after a chest x‐ray found a right pleural effusion during a physical review (Figure 1A). He had no symptoms and he had a smoking history of 40 pack‐years. Chest computed tomography (CT) demonstrated a nodular shadow bordering the pleura and right pleural effusion (Figure 1B,C). Three months later, his consultation was terminated because the pleural effusion had apparently resolved on chest x‐ray (Figure 2). He later returned when a chest x‐ray demonstrated an abnormal shadow in the right upper pulmonary lung field (Figure 3A). Chest CT found multiple, nodular papillary growths in the right pleura, but did not find pleural effusion (Figure 3B,C). Epithelioid malignant pleural mesothelioma was diagnosed on the basis of surgical lung biopsy findings. Because he lived near an asbestos factory as a child, his doctor needed to be vigilant against malignant pleural mesothelioma.

In the advanced stages of malignant pleural mesothelioma, pleural effusion may occasionally improve when tumour cells cause adhesion of the pleura.^1^ However, in the present case, the pleural effusion improved at an early stage of the disease. The patient had a benign nodule and pleural effusion caused by asbestos, but it was considered possible that the nodule became malignant and turned into pleural mesothelioma. Pleural effusion with nodular shadows bordering the pleura should be followed up even if the pleural effusion improves.

## AUTHOR CONTRIBUTIONS

Sayaka Nishida and Kazutoshi Toriyama collected the clinical data and drafted the original manuscript. Makiko Yomota and Yukio Hosomi were in charge of patient care, obtained informed consent from the patient and revised the original manuscript. All authors have confirmed the final manuscript and agreed to publication.

## CONFLICT OF INTEREST STATEMENT

None declared.

## ETHICS STATEMENT

The authors declare that appropriate written informed consent was obtained for the publication of this manuscript and accompanying images.

## Data Availability

The data that support the findings of this study are available from the corresponding author upon reasonable request.
